# Quantitative models for accelerated protein dissociation from nucleosomal DNA

**DOI:** 10.1093/nar/gku719

**Published:** 2014-08-11

**Authors:** Cai Chen, Ralf Bundschuh

**Affiliations:** 1 Biophysics Graduate Program, The Ohio State University, Columbus, OH, USA; 2 Departments of Physics and Chemistry & Biochemistry and Division of Hematology, The Ohio State University, Columbus, OH, USA; 3 Center for RNA Biology, The Ohio State University, Columbus, OH, USA

## Abstract

Binding of transcription factors to their binding sites in promoter regions is the fundamental event in transcriptional gene regulation. When a transcription factor binding site is located within a nucleosome, the DNA has to partially unwrap from the nucleosome to allow transcription factor binding. This reduces the rate of transcription factor binding and is a known mechanism for regulation of gene expression via chromatin structure. Recently a second mechanism has been reported where transcription factor off-rates are dramatically increased when binding to target sites within the nucleosome. There are two possible explanations for such an increase in off-rate short of an active role of the nucleosome in pushing the transcription factor off the DNA: (i) for dimeric transcription factors the nucleosome can change the equilibrium between monomeric and dimeric binding or (ii) the nucleosome can change the equilibrium between specific and non-specific binding to the DNA. We explicitly model both scenarios and find that dimeric binding can explain a large increase in off-rate while the non-specific binding model cannot be reconciled with the large, experimentally observed increase. Our results suggest a general mechanism how nucleosomes increase transcription factor dissociation to promote exchange of transcription factors and regulate gene expression.

## INTRODUCTION

In eukaryotes, DNA is repeatedly wrapped around histone protein complexes to form nucleosomes. This process compacts long DNA molecules so they fit inside the cell nucleus (1). Wrapping DNA into nucleosomes prevents access for DNA-binding proteins (such as transcription factors, RNA and DNA polymerases and DNA repair enzymes) to their target sites (2,3). By controlling access to DNA, nucleosomes appear to regulate many biological processes including gene expression, DNA replication, and DNA repair (4).

In order to accommodate numerous DNA processing complexes, nucleosomes undergo dynamic changes where nucleosomal DNA transiently unwraps exposing it to DNA-binding proteins by thermal fluctuations (5,6). The equilibrium between partially wrapped and fully wrapped DNA is largest near the two DNA entry–exit regions of the nucleosome (5–7). These dynamics have been quantitatively described and modeled using a free energy landscape for DNA unwrapping from a nucleosome on the 601 positioning sequence (8).

Transcription factors (TFs) are essential molecules that regulate gene expression at the transcriptional level (9). Many of their target sites fall into the entry–exit regions of nucleosomes (10) and are thus wrapped around the histone octamer. In such cases, the transcription factor is sterically occluded from its target site, which reduces the rate of transcription factor binding (11). The resulting change in transcription factor binding site occupancy is well known as a mechanism for regulation of gene expression via chromatin structure (2,3). Interestingly, recent single molecule experiments (12) found that transcription factor dissociation rates from target sites within nucleosome entry–exit regions are enhanced by two to three orders of magnitude relative to naked DNA. However, the detailed physical mechanisms are unknown.

Here, we quantitatively model this process to understand potential mechanisms behind this nucleosomal increase in TF dissociation rate. In the experiments by Luo *et al.* (12), the TF binding site is located such that it faces the histone core, i.e. such that in the bound state the TF has to be located in between the unwrapped DNA and the histone core. Thus, steric or electrostatic interactions between the TF and the nucleosome occur far away from the DNA binding site of the TF and given the size of the TF would likely result in forces that are relatively tangential to the DNA. Also, the DNA bound by the TF has to be fully unwrapped from the histone core in this geometry and thus deformations of the DNA inflicted by the TF binding can influence the DNA–histone interactions at most through hydrodynamic effects. For any of these interactions, it is difficult to imagine that they affect dissociation of transcription factors by two to three orders of magnitude. However, even if the effects of these direct interactions between the TF and the histones are to be included, understanding the mechanisms in the absence of direct forces between the nucleosome and the TF provides an important baseline.

We propose two mechanisms behind the acceleration in TF dissociation and explicitly model both of them: (i) Both TFs studied in Luo *et al.* (12), LexA and Gal4, bind DNA as a homodimer and bind to their operator half-sites with 1000-fold lower affinity (13,14). Thus, the nucleosome can change the equilibrium between monomeric and dimeric binding, a process that has also recently been pointed out to increase off-rates of dimeric transcription factors due to high concentrations of transcription factors in solution (15). (ii) The nucleosome can change the equilibrium between binding at the specific binding site and non-specific binding to the surrounding DNA. We quantitatively model both scenarios. Since the exact value of the dissociation rate from naked DNA was too slow to be experimentally determined in Luo *et al.* (12), and the binding affinity for Gal4 monomer is also not known (both are required for our modeling), we focused on the LexA case. However, while the precise parameters are not known, we expect qualitatively similar results in the Gal4 case.

Our results indicate that the non-specific binding mechanism cannot be reconciled with experimental findings, but the dimer mechanism can account for increases in off-rate by a factor as high as 200. Overall, our results suggest a general mechanism how nucleosomes increase dimeric transcription factor dissociation to facilitate transcription factor exchange and regulate gene expression.

## MATERIALS AND METHODS

We model the process that TFs dissociate from a single nucleosome since experimentally the presence of neighboring nucleosomes does not affect the association or dissociation rates (12). In both of our models, the dynamics are summarized by rate matrices, which satisfy the master equation:

\begin{equation*} \frac{d}{{dt}}X(t) = R\cdot X \end{equation*}

where $X = \left( {\begin{array}{*{20}c} {X_1 (t)} \\ {...} \\ {X_n (t)} \\\end{array}} \right)$ and *X_i_*(*t*) are the time-dependent concentrations of each state (*n* is the total number of states).

The solution to this master equation is

\begin{equation*} X(t) = C_1 \exp (\lambda _1 t) + C_2 \exp (\lambda _2 t) + ... + C_n \exp (\lambda _n t) \end{equation*}

where $C_i = \left( {\begin{array}{*{20}c} \begin{array}{l} X_{i1} \\ X_{i2} \\ \end{array} \\ {...} \\ {X_{in} } \\\end{array}} \right)(i = 1,2,...n)$ are the eigenvectors of ***R***, and $\lambda _i$ are the eigenvalues of ***R*** (16).

For a rate matrix, the single eigenvalue corresponding to the steady state of the system is zero, while all the remaining eigenvalues are negative. If we thus set $\lambda _1 = 0$, we get $X(t) = C_1 + C_2 \exp ( - \left| {\lambda _2 } \right|t) + ... + C_n \exp ( - \left| {\lambda _n } \right|t)$, where *C*_1_ is the steady state of the system. Therefore, the largest non-zero eigenvalue yields the slowest exponential decay rate, which dominates the approach to the steady state for long times and thus corresponds to the experimentally measured off-rate.

In both models, we first model the case that the transcription factor dissociates from naked DNA (without a nucleosome). We fit these models of binding to naked DNA to experimental data (TF off rate from naked DNA) to determine the kinetic parameters of the TF-DNA interaction.

We then incorporate the nucleosome free energy landscape data from (8) to calculate the TF off-rate in the presence of a nucleosome using the nucleosome unwrapping and rewrapping rates. This calculated off-rate is compared with the experimental values. In general, the nucleosome unwrapping and rewrapping rates for the *n*th base pair of DNA are determined by:

\begin{equation*} \begin{array}{l} k_{{\rm unwrap}} = k_0 e^{ - (\Delta G_{{\rm nuc}} (n) - \Delta G_{{\rm nuc}} (n - 1))/k_B T} \\ k_{{\rm rewrap}} = k_0 \\ \end{array} \end{equation*}

where Δ*G*(*n*) is the Gibbs free energy landscape due to unwrapping *n* base pairs (8), *k_B_* is Boltzmann's constant and *T* is the temperature. We take *k*_0_ = 10^5^ s^−1^ (the base rate for wrapping fluctuations) from (8). In the dimeric binding model, which includes a fully unwrapped, a partially unwrapped and a fully wrapped state of the nucleosome, these step-by-step unwrapping and rewrapping rates were combined into rates of changes of the number of wrapped base pairs by larger amounts at once as described in the Results section.

## RESULTS

### Dimeric binding

**Figure 1.**
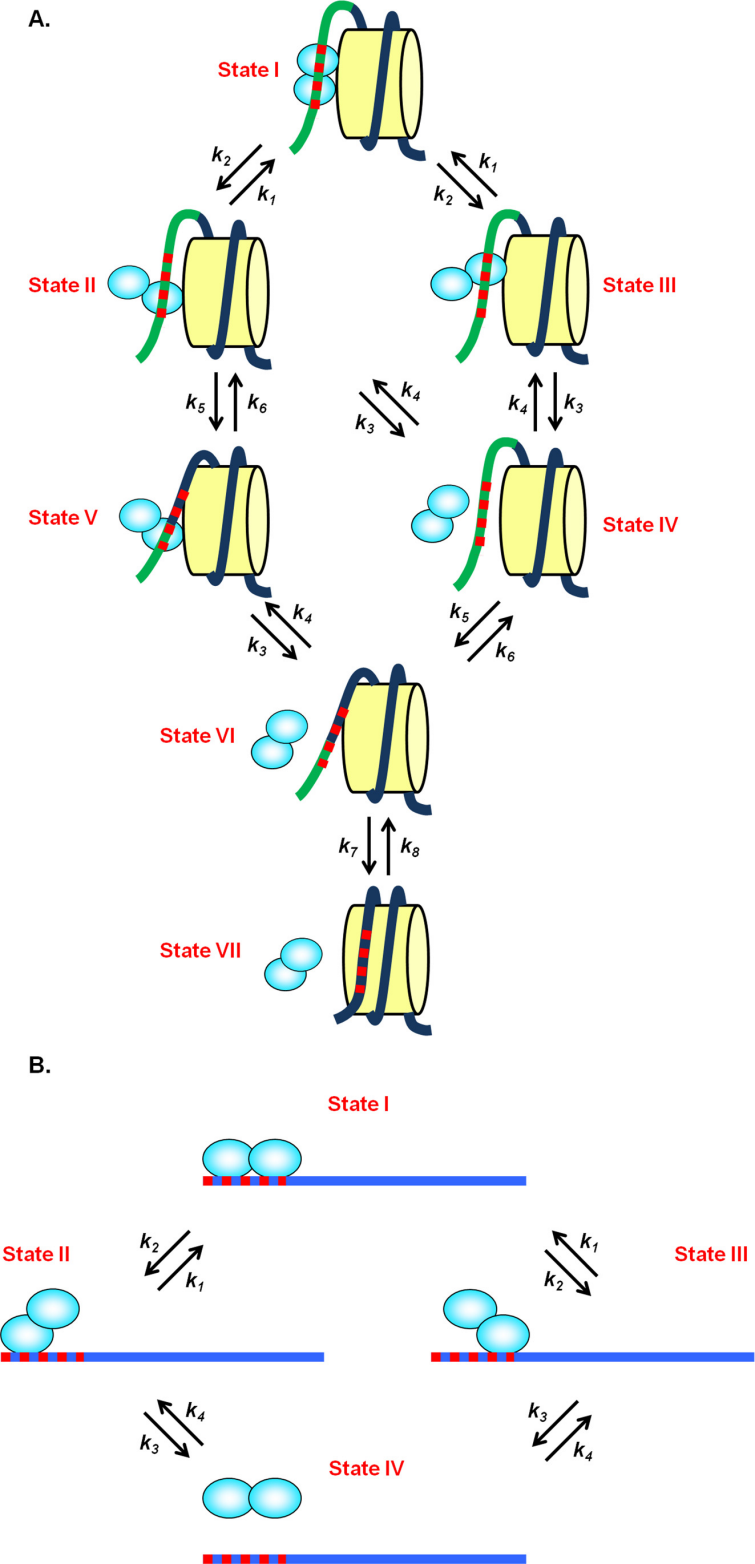
Dimeric model of TF dissociation from (A) a nucleosome and (B) from naked DNA. (A) In the presence of a nucleosome (yellow cylinder), if the site which is closer to the nucleosome dyad dissociates first (State I to State II), the nucleosome can rewrap (State II to State V). Once the other site dissociates, the TF will fall off (State V to State VI) and the nucleosome will be completely wrapped (State VI to State VII). If the further site dissociates first (State I to State III), the nucleosome needs to wait for the closer site to dissociate and then be rewrapped (State III to VII). The dark blue line is the DNA that is wrapped around the histone core while the light green line is the DNA unwrapped from the nucleosome. The red dashed line is the TF binding site. (B) In the absence of the nucleosome, when one of two binding sites dissociates (State I to State II or III), it can rebind again (State II or III to State I) or completely dissociate (State II or III to State IV).

In the study of Luo *et al.* (12), two transcription factors were investigated: (i) *Escherichia coli* transcription repressor protein LexA, which has a very specific binding site of 16 bp and dissociation constant of *K_d_* ∼ 0.1 nM (17), and forms a stable dimer where each monomer binds to a half site of the whole LexA binding site (13,18,19); (ii) A model eukaryotic transcription factor Gal4, which recognizes its 17 bp consensus binding site with an even smaller dissociation constant of *K_d_* ∼ 10 pM (20) and also binds DNA as a homodimer (20,21). Since both TFs are homodimers and bind to two specific half sites, we propose that one of the two binding sites will dissociate first. In the presence of a nucleosome (Figure 1A), if the site that is closer to the nucleosome dyad dissociates first, the nucleosome can rewrap and prevent rebinding. Once the other site dissociates, the TF will completely detach. Therefore, when the nucleosome is present it can ‘rachet’ the protein off by blocking reassociation at the site proximal to it. However, in the absence of the nucleosome, when one of two binding sites dissociates, it would rapidly rebind again, meaning that both sites dissociating at once would be rare. This should increase the off-rate of the TF in the presence of a nucleosome relative to naked DNA, just as it does in the presence of multiple dimeric non-specific binding proteins saturating on a stretch of DNA (15).

Here, we model this process quantitatively to test if this model can explain the experimental results of Luo *et al.* (12). To determine the rates of transition between the partially bound states in the presence of a nucleosome, we first build our model for the case that the TF binds to naked DNA. This model for naked DNA consists of four states (Figure 1B), namely, both TF half sites unbound, only one of the two half sites bound and both half sites bound.

To describe the transitions in this model, we introduce the rates *k*_1_, *k*_2_, *k*_3_ and *k*_4_: *k*_1_ is the transition rate from either of the partially bound states to the fully bound state (both half sites bound), while *k*_2_ is the rate from the fully bound state to either of the partially bound states. *k*_3_ is the transition rate from either partially bound state to the fully unbound state while *k*_4_ is the rate for the TF to bind to one of the half sites.

These dynamics can be summarized by the rate matrix

\begin{equation*} R = \left( {\begin{array}{*{20}c} { - 2k_2 } & {k_1 } & {k_1 } & 0 \\ {k_2 } & { - (k_1 + k_3 )} & 0 & {k_4 } \\ {k_2 } & 0 & { - (k_1 + k_3 )} & {k_4 } \\ 0 & {k_3 } & {k_3 } & { - 2k_4 } \\ \end{array}} \right) \end{equation*}

We have to determine the values of *k*_1_, *k*_2_, *k*_3_ and *k*_4_ from experiment, at which point we have to limit ourselves to LexA as indicated in the introduction. Since experiments provide only a measurement of the overall dissociation rate of the TF, we first need to find additional relations between the rates. Based on the results in (13), when one of the two half sites is mutated (or when the dimerization domain of LexA is removed), LexA can still bind to the half site that is not mutated, but with ∼1000-fold lower affinity. If we call the dissociation constant of LexA fully bound *K_d_*_(LexA, full)_ and the dissociation constant of LexA partially bound (LexA only binds to a half site) *K_d_*_(LexA, partial)_, we thus have *K_d_*_(LexA, full)_ / *K_d_*_(LexA, partial)_ = 1/1000. Since *K_d_*_(LexA, full)_ / *K_d_*_(LexA, partial)_ = exp (-$\Delta G/k_B T$) = *k*_2_ / *k*_1_, where $\Delta G$ is the free energy difference between a partially bound and the fully bound state, we get *k*_1_ / *k*_2_ = 1000. Moreover, since both *k_2_* and *k_3_* represent how fast one of the LexA monomers dissociates from the DNA, we treat these two rates to have the same numerical value (*k*_2_ = *k*_3_). Then, to calculate the overall off rate of LexA, we set *k*_4_ = 0 to designate the state, in which LexA is unbound, the steady state and solve for the largest non-zero eigenvalue of the rate matrix ***R***. This eigenvalue can be calculated analytically to be

\begin{equation*} \frac{1}{2}[(k_1 + 2k_2 + k_3 ) - \sqrt {(k_1 + 2k_2 + k_3 )^2 - 8k_2 k_3 } ].\end{equation*}

By equating the absolute value of this overall off rate to the experimental value of 0.0034 s^−1^ (12) and using the relationships *k*_2_ = *k*_3_ and *k*_1_ / *k*_2_ = 1000 introduced above, we can solve for *k*_1_, *k*_2_ and *k*_3_ and obtain *k*_1_ = 1700 s^−1^ and *k*_2_ = *k*_3_ = 1.7 s^−1^. *k*_4_ can then be inferred from measurements of the overall *K_d_*_(LexA, full)_ (∼0.07 nM) which yields *k*_4_ = 0.025 s^−1^ nM^−1^ [LexA] where [LexA] denotes the concentration of LexA.

In the presence of a nucleosome, if the binding site closer to the nucleosome dyad dissociates first, the nucleosome can rewrap thus facilitating TF dissociation (Figure 1A). Keeping track of the nucleosome unwrapping state leads us to a model with seven states (Figure 1A). In this model, the transition rates *k*_1_, *k*_2_, *k*_3_ and *k*_4_ are only dependent on the TF and its binding target and are completely unrelated to the nucleosome; we thus use the same values for these four parameters as in the naked DNA case. We further introduce the rates *k*_5_, *k*_6_, *k*_7_ and *k*_8_, which reflect the nucleosome rewrapping and unwrapping. Since the TF will only affect the properties of the nucleosomal DNA that is already unwrapped for TF binding (not related to *k*_5_, *k*_6_, *k*_7_ and *k*_8_) but not the nucleosomal DNA still wrapped around the histone core (related to *k*_5_, *k*_6_, *k*_7_ and *k*_8_), the TF can affect the wrapping and rewrapping rates at most through changing the hydrodynamics of the unwrapped DNA (other than the obvious effect that the binding of the TF entirely prevents certain wrapping events, which we explicitly include here through the structure of our model). We thus assume that the rates *k*_5_, *k*_6_, *k*_7_ and *k*_8_ are not affected by the presence of the TF and take them from (8) as

\begin{equation*} \begin{array}{l} k_5 = k_0 /(n_f - n_p ) \\ k_7 = k_0 /(n_p - n_w ) \\ k_6 = k_5 e^{ - [\Delta G(n_f ) - \Delta G(n_p )]/k_B T]} \\ k_8 = k_7 e^{ - [\Delta G(n_p ) - \Delta G(n_w )]/k_B T]} \\ \end{array} \end{equation*}

where *n_f_* is the number of DNA base pairs unwrapped from the nucleosome that is required for the TF to fully bind (states I, II, III and IV in Figure 1A), *n_p_* is the number of DNA base pairs unwrapped that is required for the TF to bind to the half site that is farther from the nucleosome core (states V and VI in Figure 1A), and *n_w_* is 0 which represents the number of unwrapped base pairs when the nucleosome is fully wrapped (state VII in Figure 1A). The rate *k*_5_ describes rewrapping from states that allow the TF to bind fully (states I, II, III and IV in Figure 1A) to states that allow only partial binding of the TF (states V and VI in Figure 1A). This rewrapping comprises (*n_f_* - *n_p_*) independent and individual rewrapping steps in sequence. Thus, the rewrapping time of this entire event is (*n_f_* - *n_p_*) times the rewrapping time of an individual base pair, resulting in the rewrapping rate to be 1/(*n_f_* - *n_p_*) of the base rate of *k*_0_ = 10^5^ s^−1^ for wrapping fluctuations from (8) (see Materials and Methods section). Analogous arguments yield the effective rewrapping rate *k*_7_.

Similar to the rate matrix in the absence of a nucleosome, the rate matrix for the seven state model with a nucleosome can be written as

\begin{equation*}
\left( {\begin{array}{*{20}c} { - 2k_2 } & {k_1 } & {k_1 } & 0 & 0 & 0 & 0 \\ {k_2 } & { - (k_1 + k_3 + k_5 )} & 0 & {k_4 } & {k_6 } & 0 & 0 \\ {k_2 } & 0 & { - (k_1 + k_3 )} & {k_4 } & 0 & 0 & 0 \\ 0 & {k_3 } & {k_3 } & { - (2k_4 + k_5 )} & 0 & {k_6 } & 0 \\ 0 & {k_5 } & 0 & 0 & { - (k_3 + k_6 )} & {k_4 } & 0 \\ 0 & 0 & 0 & {k_5 } & {k_3 } & { - (k_4 + k_6 + k_7 )} & {k_8 } \\ 0 & 0 & 0 & 0 & 0 & {k_7 } & { - k} \\ \end{array}_8 } \right).
\end{equation*}

For LexA, all its parameters are determined either by the naked DNA experiments or from (8). In the experiment, only the state in which LexA completely dissociates from the nucleosome and the nucleosome is fully wrapped (state VII in Figure 1A) is detected as the ‘off’ state based on its high FRET signal. Therefore, to calculate the overall off rate of LexA in the presence of a nucleosome, we set *k*_8_ = 0 to designate this high FRET state as the steady state (this also implies that Δ*G*(*n_p_*)- Δ*G*(*n_w_*) is not relevant for the off-rate).

**Figure 2.**
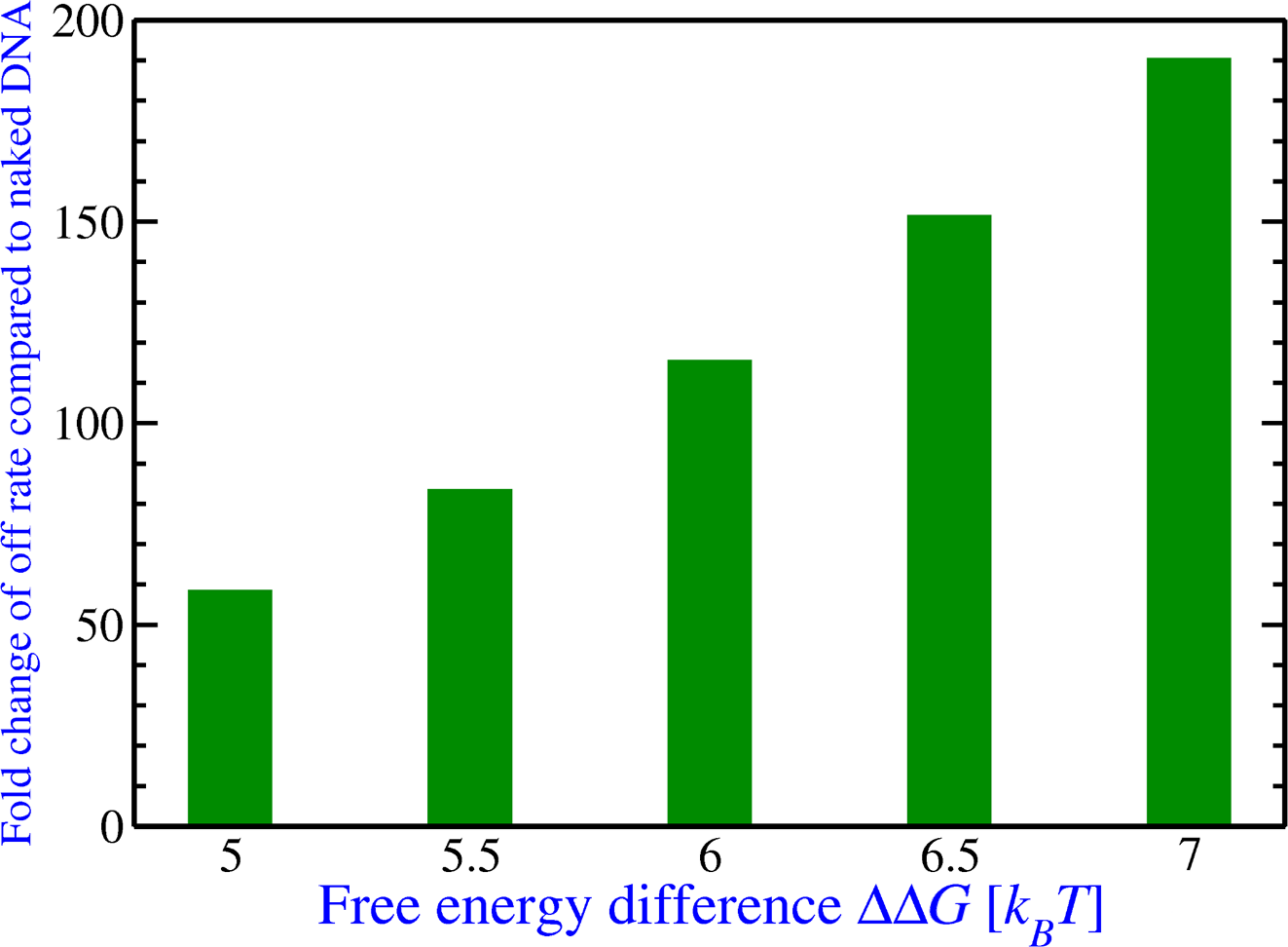
The overall off-rate of LexA dissociation from the nucleosome compared to naked DNA for the dimeric model. Several numerical values of the nucleosome unwrapping free energy difference between State II and State IV of the model in Figure 1A are used to calculate the overall off rate of LexA in the presence of a nucleosome which is shown here as a fold change compared to the naked DNA case.

The overall off-rate of LexA in the presence of a nucleosome can be calculated as the largest non-zero eigenvalue of the rate matrix of the seven state model numerically (22). In our calculation, we found that the only parameter the overall off rate is sensitive to is the free energy difference ΔΔ*G* ≡ Δ*G*(*n_f_*) - Δ*G*(*n_p_*) between the two states when LexA is fully or partially bound (Supplementary Text). For example, the concentration of LexA affects the overall off rate (*k*_4_ is not equal to zero in the presence of a nucleosome); however, we tested a number of different LexA concentrations ranging from 0 to 50 000 nM as also tested in the experiment (12) and found that the overall off-rate only changes slightly (Supplementary Text). The value of the one important parameter ΔΔ*G* depends on the exact amount of unwrapping (*n_f_* and *n_p_*) of the nucleosome which is unknown. Thus, we tested several numerical values of ΔΔ*G* (5 to 7 *k_B_T*) in the range of reasonable amounts of unwrapping. We found that *k*_off_ _(nucleosome)_ ranges from 0.2 to 0.65 per second (Figure 2 and Supplementary Text), which is an increase by 50- to 200-fold compared to the naked DNA case.

### Non-specific binding

Transcription factors bind to their specific target site on DNA. However, at high concentrations, transcription factors also bind to non-specific DNA targets with much lower binding affinity. Moreover, in the process of searching for a specific target, it is inevitable that transcription factors encountering non-specific DNA scan along this non-specific DNA which affects the specific-DNA targeting rate of the protein. Studies on several DNA binding proteins including LacI revealed that proteins bind to their targets much faster than the 3D diffusion limit, which was explained by this facilitated-diffusion model (23–27). We propose that if TFs can also bind to non-specific binding sites, then the nucleosomes can rewrap and prevent rebinding of TFs to their specific binding site. Thus, nucleosomes can change the equilibrium between binding at the specific binding site and non-specific binding to the surrounding DNA; we propose that non-specific binding may contribute to the large increase of the overall off-rate of TFs in the presence of a nucleosome compared to naked DNA.

In our model, we assume that the TF can slide—diffusing in one dimension over the DNA molecule—and dissociate from any position. We model this diffusion as base-by-base diffusion along the DNA with multiple non-specific binding sites, in which the TF slides only one single base in one step.

**Figure 3.**
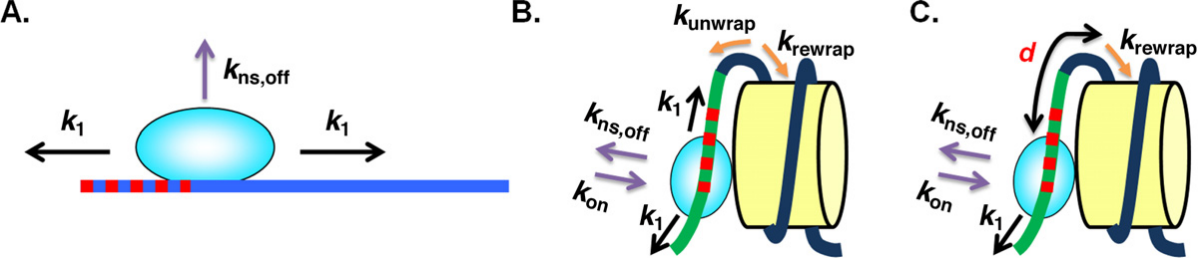
Non-specific binding model of TF dissociation from (A) naked DNA and (B and C) from a nucleosome. (A) In the absence of the nucleosome, the TF can slide along the DNA in two directions (black arrows, *k*_1_) or dissociate from the DNA (Purple arrow, *k*_ns,off_). (B) In the presence of a nucleosome, five possibilities for the next step of a certain state exist: (i) and (ii) the TF can slide along both directions in the unwrapped nucleosome (black arrows, *k*_1_); (iii) and (iv) the nucleosome can unwrap and rewrap (orange arrows, *k*_unwrap_, *k*_rewrap_); or (v) the TF can dissociate from the unwrapped nucleosome (purple arrow, *k*_ns,off_). The figure shows LexA binding to a non-specific site. Among all the binding sites, only one site is the specific target for the TF (red dashed line in (A) and (B)) and the others are non-specific binding sites for the TF. (C) Illustration of the minimum distance (d in bp) between the TF and the unwrapping position of the nucleosome. At this minimum distance when the TF binds to DNA, the nucleosome cannot rewrap anymore and the TF can only slide in one direction.

Analogously to the dimeric binding model, we first build this model in the case that the TF binds to naked DNA. Here, the TF can slide along the DNA in two directions and can also dissociate from the DNA (Figure 3A). Among all the binding sites, only one site is the specific target for the TF and the others are non-specific binding sites for the TF.

We introduce the sliding rates of the TF, *k*_1_ and *k*_2_: *k*_1_ is the sliding rate of the TF from a non-specific binding site to another site (both specific binding site and non-specific binding site), while *k*_2_ is the sliding rate of the TF from the specific binding site to a neighboring non-specific binding site. We define the ratio of *k*_1_ and *k*_2_ as ***K*** = *k*_1_ / *k*_2_. Similar to the dimeric binding model, this ratio is determined by how ‘tightly’ the TF binds to the sites, i.e. by the binding affinity difference between non-specific and specific binding sites, or the ratio of the dissociation constants between non-specific and specific binding, as

\begin{equation*} {\boldsymbol K} = k_1 /k_2 = {\boldsymbol K}_{d\,({\rm non}{-}{\rm specific}\,{\rm binding})} /{\boldsymbol K}_{d\,({\rm specific}\,{\rm binding})} .\end{equation*}

Since the TF can dissociate from any binding site, we further introduce rate parameters for TF dissociation from the specific binding site (*k*_s__,off_) and the non-specific binding sites (*k*_ns__,off_). Similarly, the ratio *k*_s__,off_ / *k*_ns,off_ is determined by the ratio of dissociation constants between non-specific and specific binding (***K*** = *k*_ns,off_ / *k*_s,off_ = *K_d_*_(non-specific binding)_ / *K_d_* _(specific binding)_). We assume that the on rate for TF reassociation to the nucleosome is the same for specific and non-specific binding sites (*k*_ns,on_ = *k*_s,on_ = *k*_ns,off_ / *K_d_*_(non-specific binding)_ · [TF] = *k*_s,off_ / *K_d_*_(specific binding)_ · [TF]).

**Figure 4.**
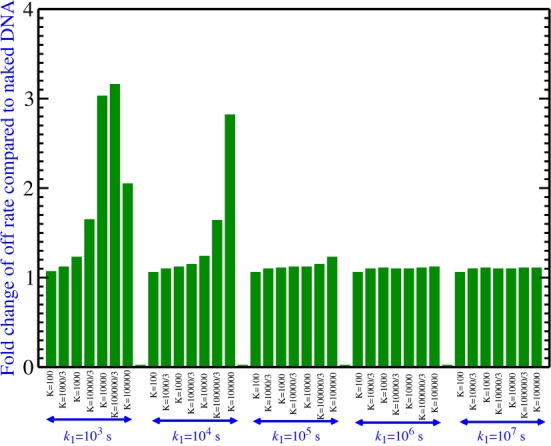
The overall off rate of LexA dissociation from a nucleosome compared to naked DNA for the non-specific binding model. The overall off rate of LexA in the presence of a nucleosome for 35 combinations of the unknown parameters ***K*** and *k*_1_ covering the entire range of biologically plausible values is calculated and presented as a fold change compared to the naked DNA case.

Again, we use the measured overall off rate of TF dissociation from naked DNA to determine the unknown parameters and thus have to limit ourselves to the case of LexA. Since several parameters are unknown, we vary their values within experimentally reasonable values. The parameters we vary are the ratio ***K*** = *K_d_*_(non-specific binding)_ /*K_d_*_(specific binding)_ and sliding rate *k*_1_ because all other parameters can be obtained based on these two. We varied ***K*** from 100 to 100,000 based on experimental estimates of this number (28), and varied *k*_1_ from 10^3^ s^−1^ to 10^7^ s^−1^ to cover the range of sliding rates of many known DNA-binding proteins (29). We investigated all combinations of ***K*** and *k*_1_ resulting in 35 different conditions (Figure 4).

For each value of ***K*** and *k*_1_ there is only one unspecified parameter (*k*_s,off_ or *k*_ns,off_, which are connected via *k*_ns,off_ = ***K*** * *k*_s,off_) that we fit by requiring that the largest non-zero eigenvalue of the rate matrix reproduce the experimentally measured overall LexA off rate from naked DNA of 0.0034 s^−1^.

These fitting parameters are then used to calculate the overall off-rate of LexA in the presence of a nucleosome for every combination of ***K*** and *k*_1_ explored. In the presence of a nucleosome (Figure 3B), LexA can (i) slide along both directions in the unwrapped nucleosome; or (ii) dissociate from or reassociate to the unwrapped nucleosome. In the meantime, the nucleosome can unwrap and rewrap and this can affect LexA binding and dissociation. Similar to the dimeric model we can calculate the base-by-base nucleosome rewrapping and unwrapping rates (Materials and Methods), which then specifies the rate matrix. Similar to the dimeric model only the state in which LexA dissociates from the nucleosome and the nucleosome is fully wrapped is considered as the ‘off’ state (high FRET state in the experiment).

Due to the unknown bulk size of the proteins, we further define a parameter *d* that is the minimum distance (in *bp*) between LexA and the unwrapping position of the nucleosome. At this minimum distance when LexA binds to DNA, the nucleosome cannot rewrap anymore and the LexA protein can only slide in one direction (Figure 3C). This parameter affects the off-rate of the TF because it determines which particular point in the nucleosome free energy landscape corresponds to the sliding of the TF onto and off of its specific binding site; since the nucleosome free energy landscape is not simply a straight line of constant slope (8), this position determines the unwrapping rate associated with the specific binding site and thus directly influences the dynamics of the system. For each ***K*** and *k*_1_, we tested *d* = 0, 1, …, 21 and picked the *d* with the largest off-rate among all 22 largest non-zero eigenvalues in order to obtain an upper bound for the increase of the overall off-rate in the presence of a nucleosome compared to the naked DNA. We found that in all the ***K*** and *k*_1_ we tested, we always got the largest off-rate when *d* = 7 (i.e. when the minimum distance between LexA and the unwrapping position of the nucleosome is 7 bp) (Supplementary Table S1). Similarly, we varied [LexA] and found the largest off-rates at [LexA] = 0 nM (Supplementary Table S2, note that we started with the state in which one LexA molecule is fully bound to the DNA even in the absence of LexA in solution). Nevertheless, in spite of choosing the maximizing values of *d* and [LexA], the off-rates for none of the reasonable choices of ***K*** and *k*_1_ differ significantly from the overall off-rate of LexA from naked DNA (0.0034 s^−1^) (Figure 4 and Supplementary Table S3).

In order to investigate the sensitivity of our finding on the specifics of the nucleosome unwrapping free energy landscape, we also tested the effect of changing the position of the LexA binding site from positions 8–27 within the 601 nucleosome positioning sequence (used in the experiment of Luo *et al.* (12)) to 1–20 and 18–37. However, even then we found increases in the overall off-rate of LexA by at most one order of magnitude compared to naked DNA (Supplementary Table S4).

Based on these results, we conclude that the non-specific binding model cannot explain the large increase of the overall off rate of LexA in the presence of a nucleosome compared to naked DNA as observed in the experiment (12). It might be true that LexA does not slide on DNA (29) as there is no such experimental evidence; however, even if it slides on DNA, it is not the sliding that can explain the large increase of its off-rate from a nucleosome compared to the naked DNA based on the non-specific model.

### Predictions for future experiments

In addition to explaining the experimental results of Luo *et al.* (12), we can now use our dimeric binding model to make predictions that can be tested in future experiments.

First, we investigate the consequences of mutating away half of the LexA binding site, just as done for naked DNA in (13). This includes two cases: (i) mutating the half site near the nucleosome dyad; (ii) mutating the half site far from the nucleosome dyad. We model both cases following the dimeric binding model and using the same parameters choosing ΔΔ*G* = 6 *k_B_T*. Here, the LexA fully bound state no longer exists and the seven states reduce to three as indicated for each case in Supplementary Figure S1. We find that in both cases the overall off-rate of LexA dissociation from the nucleosome is approximately equal to *k*_3_ (∼1.7 s^−1^). Unlike the non-mutated case, it is not sensitive to the free energy differences between the fully and partially unwrapped state of the nucleosome. While this value (∼1.7 s^−1^) is a 500-fold increase compared to the overall off-rate of LexA from naked DNA without a mutation and a 5-fold increase compared to the overall off rate of LexA from a nucleosome predicted from the dimeric binding model, it is indeed the same as the off rate *k*_3_ from naked DNA with one half site mutated. This indicates that the overall off-rate of LexA in the presence of the nucleosome should not be changed compared to naked DNA when half of its target is mutated—a result that also applies to a TF that binds as a monomer.

Furthermore, since many TFs bind close to the entry–exit region, we investigated the case when half of the LexA binding site is located outside of the nucleosome positioning sequence (from −10 to −1) such that the nucleosome cannot unwrap or rewrap in this region, while the other half is within the nucleosome positioning sequence (from 1 to 10) (Supplementary Figure S2). Similarly, we model this process with the dimeric binding model using the same parameters but changing nucleosome unwrapping and rewrapping states and rates based on (8). We find that the overall off-rate of LexA dissociation from the nucleosome is ∼1.45 s^−1^, a 400-fold increase compared to the overall off-rate of LexA from naked DNA and a 4-fold increase compared to the overall off-rate of LexA from within the nucleosome at the same parameters.

## DISCUSSION

In this work, we quantitatively modeled the process of transcription factor dissociation from a nucleosome by investigating two possible mechanisms that could explain a recent single-molecule study. In the study of Luo *et al.* (12), they reported that the dissociation rates of two transcription factors, LexA and Gal4, from a target site within the nucleosome entry–exit region are enhanced by two to three orders of magnitude relative to naked DNA. We choose LexA as a model because some of the experimental data required for our modeling are not available for Gal4. However, the results should be very similar for Gal4 as the two TFs share similar properties, such as that both of them bind long and specific DNA binding sites (the LexA binding site is 16 bp long (18) while the Gal4 binding site is 17 bp long (21)) as homodimers. Since a large number of eukaryotic TFs can bind to their target sites in a dimeric form (in human, at least one quarter of the TFs bind to their target sites as dimers—the best studied TFs that can form dimers alone number at least 500 (30), while the upper bound on the number of TF-coding genes is 1700–1900 (31)), our dimeric binding model is applicable to a wide range of eukaryotic TFs and not just the two specific ones used in the experiments by Luo *et al.* (12).

We first modeled the case that LexA dissociates from naked DNA (without a nucleosome). We fit these models to experimental data to determine the kinetic parameters of transcription factor-DNA interaction. We then incorporated nucleosome free energy landscape data from (8) to calculate the LexA off-rate in the presence of a nucleosome. This calculated off-rate was then compared with the experimental values (12). Our results indicate that the non-specific binding mechanism cannot be reconciled with experimental findings but that the dimeric binding model can indeed explain increases in off-rate by a factor as high as 200. Finally, based on the dimeric binding model we made further predictions of other cases that are experimentally testable in the future, most specifically that the significant acceleration of dissociation should not occur if the half site is mutated away or for transcription factors that bind as monomers. Another interesting consequence of our calculations is that the off-rates of monomeric TFs should not show the dramatic increase observed for dimeric TFs, since monomeric TFs would be described by the non-specific binding model. It would be interesting to see this prediction tested in future experiments as well.

It is important to point out that the 601 nucleosome positioning sequence, which was used in the experiment and in the determination of the free energy landscape (8) on which our calculations are based, has an unusually high binding affinity to the histone much stronger than what occurs *in vivo*. Thus, the question arises if our calculations (and the experimental results they explain) are specific to the 601 sequence. In fact, since it is not known precisely which bases unwrap due to the binding of the TF, our calculations of the dimeric model never refer to specific positions in the free energy landscape, but employ the free energy landscape solely to determine reasonable values of the only relevant free energy difference ΔΔ*G* (see Figure 2). The differences in overall free energy between the 601 nucleosome positioning sequence and other less stable positioning sequences found *in vivo*, e.g. the 5S positioning sequence, are several *k_B_T* compared to an overall free energy of 30–40 *k_B_T* (8). Thus, for these less stable nucleosome positioning sequences the overall slope of the free energy landscape will be smaller than the one for the 601 sequence but not significantly smaller. This implies that, while the ΔΔ*G* for these other sequence may not reach to the upper bound of the range for the 601 sequence (∼6–7 *k_B_T*), the lower end of the range (∼5–6 *k_B_T*), which based on our calculations still yields a 50-fold increase in off-rate, is a reasonable assumption for these less stable nucleosome positioning sequences. Thus, our calculations and the experimental findings should still be applicable to weaker nucleosome positioning sequences found *in vivo* albeit with a somewhat reduced effect.

We note that although the dimeric binding model explains a large increase in the overall off-rate of LexA dissociation from a nucleosome compared to naked DNA, it still misses another 5- to 10-fold increase compared to the 1000-fold increase of the off-rate in the experimental data. This might indicate the presence of some direct steric or electrostatic force that the nucleosome exerts on LexA. Since direct nucleosome-TF interactions will be diminished if the TF is facing away from the histone, the importance of such direct interactions could in principle be tested by repeating the experiments with an outward facing TF, i.e. by moving the TF binding site 5 to 6 bp in either direction from the site in (12); however, such data are not currently available. One recent theoretical study (32) shows that nucleosomes can exert force on RNA polymerase and that this force can slow the polymerase transcribing down by about 10-fold. Another recent experimental study indicates that a neighboring nucleosome can affect the off-rate of a transcription factor from its binding site by ∼7-fold presumably through mechanical interactions (33). While this provides evidence that nucleosomes can indeed affect TF dissociation, the factor is far away from the 1000-fold changes in off-rate as observed in Luo *et al.* (12). However, these findings might give some explanation of the 5- to 10-fold increase in off-rate that our dimeric binding model is missing. In order to investigate this further, the effect of a largely tangential force on TF unbinding would have to be understood in more detail. Overall, our results suggest a general mechanism how transcription factors dissociate from nucleosomes to promote exchange of transcription factors and regulate gene expression.

## SUPPLEMENTARY DATA

Supplementary Data are available at NAR Online.

SUPPLEMENTARY DATA
