# Supplementary material for: Quantitative models for accelerated protein dissociation from nucleosomal DNA
Source: Nucleic Acids Res. 2014 Aug 11;42(15):9753–60. doi: 10.1093/nar/gku719 (PMC4150810; doi:10.1093/nar/gku719)
Supplement: SUPPLEMENTARY DATA [file supp_42_15_9753__index.html]

Quantitative models for accelerated protein dissociation from nucleosomal DNA — Quantitative models for accelerated protein dissociation from nucleosomal DNA — Quantitative models for accelerated protein dissociation from nucleosomal DNA — SUPPLEMENTARY DATA 

# *Drosophila* COP9 signalosome subunit 7 interacts with multiple genomic loci to regulate development

## SUPPLEMENTARY DATA

**Files in this Data Supplement:**

- SUPPLEMENTARY DATA
