# Supplementary material for: Quantitative models for accelerated protein dissociation from nucleosomal DNA
Source: Nucleic Acids Res. 2014 Aug 11;42(15):9753–60. doi: 10.1093/nar/gku719 (PMC4150810; doi:10.1093/nar/gku719)
Supplement: SUPPLEMENTARY DATA [file supp_gku719_nar-00618-z-2014-File006.pdf]

Supplementary Text

Effects of parameters in the dimeric model. The parameters in the dimeric model are tested in a range of reasonable values to calculate the overall off rate of LexA in the presence of a nucleosome. We find that the overall off rate is sensitive to the free energy difference $\Delta\Delta G \equiv \Delta G(n_f) - \Delta G(n_p)$ between the two states when LexA is fully or partially bound (Fig. 2) but not to others. Unless otherwise noted, all the data here are obtained when k_1 = 1700 s^{-1}, $k_2 = k_3$ = 1.7 s^{-1}, [LexA] = 0 nM (k_4 = 0 s^{-1}), k_5 = 10000 s^{-1}, $\Delta\Delta G$ = 6 k_BT (k_6 = 24.8 s^{-1}), and k_7 = 10000 s^{-1}.

Effects of free energy difference $\Delta\Delta G \equiv \Delta G(n_f) - \Delta G(n_p)$

$\Delta\Delta G \equiv \Delta G(n_f) - \Delta G(n_p)$ (k_BT)	5	5.5	6	6.5	7
$k_{off\ (nucleosome)}$ (s^{-1})	0.196	0.282	0.390	0.513	0.645
Fold changes to $k_{off\ (DNA)}$	58	83	115	151	190

Effects of LexA concentration

[LexA] (nM)	0	500	1500	5000	15000	50000
$k_{off\ (nucleosome)}$ (s^{-1})	0.390	0.389	0.389	0.386	0.378	0.354
Fold changes to $k_{off\ (DNA)}$	115	114	114	114	111	104

Effects of k_1, k_2 ($k_3 = k_2$)

k_1 / k_2	500	1000	2000	5000	10000
$k_{off\ (nucleosome)}$ (s^{-1})	0.429	0.563	0.651	0.618	0.490
Fold changes to $k_{off\ (DNA)}$	86	113	130	124	98

Effects of rewrapping rates k_5 and k_7

k_5	10000	10000	5000	5000
k_7	10000	5000	10000	5000
$k_{off\ (nucleosome)}$ (s^{-1})	0.390	0.390	0.378	0.378
Fold changes to $k_{off\ (DNA)}$	115	115	111	111

Supplementary Tables

d	$k_{off\ (nucleosome)}$ (s^{-1})	Fold change to $k_{off\ (DNA)}$
0	0.00488	1.44
1	0.00557	1.64
2	0.00758	2.33
3	0.00688	2.02
4	0.00658	1.94
5	0.00669	1.97
6	0.00761	2.24
**7**	**0.0118**	**3.47**
8	0.00927	2.73
9	0.00789	2.32
10	0.00728	2.14
11	0.00696	2.05
12	0.00695	2.04
13	0.00596	1.75
14	0.00525	1.54
15	0.00509	1.50
16	0.00497	1.46
17	0.00492	1.45
18	0.00478	1.41
19	0.00471	1.39
20	0.00473	1.39
21	0.00480	1.41

Supplementary Table 1. Effects of *d* (the minimum distance (in *bp*) between LexA and the unwrapping position of the nucleosome) in the non-specific binding model. We varied the parameter *d* (the minimum distance between LexA and the unwrapping position of the nucleosome) in the non-specific binding model to calculate the overall off rate of LexA in the presence of a nucleosome. All the data are obtained when $\boldsymbol{K}$ **=** 100000/3, k_1 = 10^3 s^{-1}, k_2 = 0.03 s^{-1}, $k_{ns,off}$ = 11.5 s^{-1}, and $k_{s,off}$ = 0.000346 s^{-1}. Base by base rewrapping and unwrapping rates are calculated based on (1). The results in the non-specific binding model are calculated when [LexA] (the concentration of LexA) is equal to zero as this gives the largest off rate compared to other concentrations (Table S2) and since we are trying to explain a large increase of the overall off rate in the presence of a nucleosome compared to the naked DNA. The value *d* =7 with the highest fold change is highlighted in bold.

[LexA] (nM)	$k_{off\ \mathrm{(nucleosome)}}$ (s^{-1})	Fold change to $k_{off\ \mathrm{(DNA)}}$
**0**	**0.0118**	**3.47**
500	0.0117	3.14
1500	0.0115	3.38
5000	0.0110	3.24
15000	0.0102	3.00
50000	0.00866	2.55

Supplementary Table 2. Effects of [LexA] (LexA concentration) in the non-specific binding model. We varied LexA concentration in the non-specific binding model to calculate the overall off rate of LexA in the presence of a nucleosome. All the data are obtained when d = 7, $\boldsymbol{K}$ **=** 100000/3**,** $k_1 = 10^3$ s^{-1}, $k_2 = 0.03\ s^{-1}$, $k_{ns,off} = 11.5\ s^{-1}$, and $k_{s,off} = 0.000346\ s^{-1}$, and $k_{ns,on} = k_{s,on} = 0.005\ s^{-1}\ nM^{-1}$ [LexA]. Base by base rewrapping and unwrapping rates are calculated based on (1). The line with [LexA] = 0 nM which yields the largest fold change is highlighted in bold.

K	k_1 (s^{-1})	k_{off} (nucleosome) (s^{-1}) (d = 7)	Fold change to k_{off} (DNA)
100	10^3	0.00360	1.06
100	10^4	0.00359	1.05
100	10^5	0.00359	1.05
100	10^6	0.00359	1.05
100	10^7	0.00359	1.05
1000/3	10^3	0.00375	1.10
1000/3	10^4	0.00368	1.08
1000/3	10^5	0.00368	1.08
1000/3	10^6	0.00368	1.08
1000/3	10^7	0.00368	1.08
1000	10^3	0.00400	1.18
1000	10^4	0.00374	1.10
1000	10^5	0.00371	1.09
1000	10^6	0.00372	1.09
1000	10^7	0.00372	1.09
10000/3	10^3	0.00489	1.44
10000/3	10^4	0.00383	1.13
10000/3	10^5	0.00374	1.10
10000/3	10^6	0.00374	1.10
10000/3	10^7	0.00372	1.09
10000	10^3	0.00827	2.43
10000	10^4	0.00404	1.19
10000	10^5	0.00377	1.11
10000	10^6	0.00374	1.10
10000	10^7	0.00374	1.10
100000/3	10^3	0.0118	3.47
100000/3	10^4	0.00488	1.43
100000/3	10^5	0.00383	1.13
100000/3	10^6	0.00375	1.10
100000/3	10^7	0.00375	1.10
100000	10^3	0.00806	2.37
100000	10^4	0.00790	2.32
100000	10^5	0.00400	1.18
100000	10^6	0.00375	1.10
100000	10^7	0.00373	1.10

Supplementary Table 3. Combinations of K (= $k_{ns,off}/k_{s,off}$ = $K_{d\ (nonspecific\ binding)}/K_{d\ (specific\ binding)}$) and k_1 used in the non-specific binding model. The overall off rate of LexA in the presence of a nucleosome for every reasonable combination of unknown parameters K and k_1 in the non-specific binding model is calculated and presented as a fold change compared to the naked DNA case reported

in Fig. 4.

(A).

K	$\boldsymbol{k_1}$ (s^{-1})	$k_{off\ \text{(nucleosome)}}$ (s^{-1}) **(*d* = 19)[a]**	Fold change to $k_{off\ \text{(DNA)}}$
100	10^3	0.000686	0.20
100	10^4	0.000683	0.20
100	10^5	0.000683	0.20
100	10^6	0.000683	0.20
100	10^7	0.000683	0.20
1000/3	10^3	0.000517	0.15
1000/3	10^4	0.000506	0.15
1000/3	10^5	0.000506	0.15
1000/3	10^6	0.000506	0.15
1000/3	10^7	0.000506	0.15
1000	10^3	0.000487	0.14
1000	10^4	0.000455	0.13
1000	10^5	0.000451	0.13
1000	10^6	0.000451	0.13
1000	10^7	0.000451	0.13
10000/3	10^3	0.000574	0.17
10000/3	10^4	0.000444	0.13
10000/3	10^5	0.000433	0.13
10000/3	10^6	0.000433	0.13
10000/3	10^7	0.000430	0.13
10000	10^3	0.00102	0.30
10000	10^4	0.000463	0.14
10000	10^5	0.000430	0.13
10000	10^6	0.000426	0.13
10000	10^7	0.000426	0.13
100000/3	10^3	0.00221	0.65
100000/3	10^4	0.000566	0.17
100000/3	10^5	0.000436	0.13
100000/3	10^6	0.000426	0.13
100000/3	10^7	0.000425	0.13
100000	10^3	0.00295	0.87
100000	10^4	0.00101	0.30
100000	10^5	0.000460	0.14
100000	10^6	0.000427	0.13
100000	10^7	0.000424	0.12

(B).

K	k_1 (s^{-1})	k_{off} (nucleosome) (s^{-1}) (d = 2)[b]	Fold change to k_{off} (DNA)
100	10^3	0.00703	2.07
100	10^4	0.00700	2.06
100	10^5	0.00700	2.06
100	10^6	0.00700	2.06
100	10^7	0.00700	2.06
1000/3	10^3	0.0133	3.91
1000/3	10^4	0.0131	3.85
1000/3	10^5	0.0131	3.85
1000/3	10^6	0.0131	3.85
1000/3	10^7	0.0131	3.85
1000	10^3	0.0223	6.56
1000	10^4	0.0219	6.44
1000	10^5	0.0218	6.41
1000	10^6	0.0218	6.41
1000	10^7	0.0218	6.41
10000/3	10^3	0.0272	8.00
10000/3	10^4	0.0300	8.82
10000/3	10^5	0.0303	8.91
10000/3	10^6	0.0304	8.94
10000/3	10^7	0.0303	8.91
10000	10^3	0.0194	5.71
10000	10^4	0.0319	9.38
10000	10^5	0.0338	9.94
10000	10^6	0.0342	10.06
10000	10^7	0.0343	10.09
100000/3	10^3	0.00950	2.79
100000/3	10^4	0.0269	7.91
100000/3	10^5	0.0334	9.82
100000/3	10^6	0.0349	10.26
100000/3	10^7	0.0354	10.41
100000	10^3	0.00644	1.89
100000	10^4	0.0169	4.97
100000	10^5	0.0290	8.53
100000	10^6	0.0327	9.62
100000	10^7	0.0339	9.97

Supplementary Table 4. Effects of the position of LexA binding sites in the non-specific binding model. (A).Effective off rates when the LexA binding site is located from position 1 to 20 within the nucleosome positioning sequence. (B). Effective off rates when the LexA binding site is located from

position 18 to 37 within the nucleosome positioning sequence.

Note:

[a] We show the results from *d* = 19 because we always get the largest off rate when varying *d* from 0 to 21 under these conditions.

[b] We show the results from *d* = 2 because we always get the largest off rate when varying *d* from 0 to 21 under these conditions.

Supplementary Figures

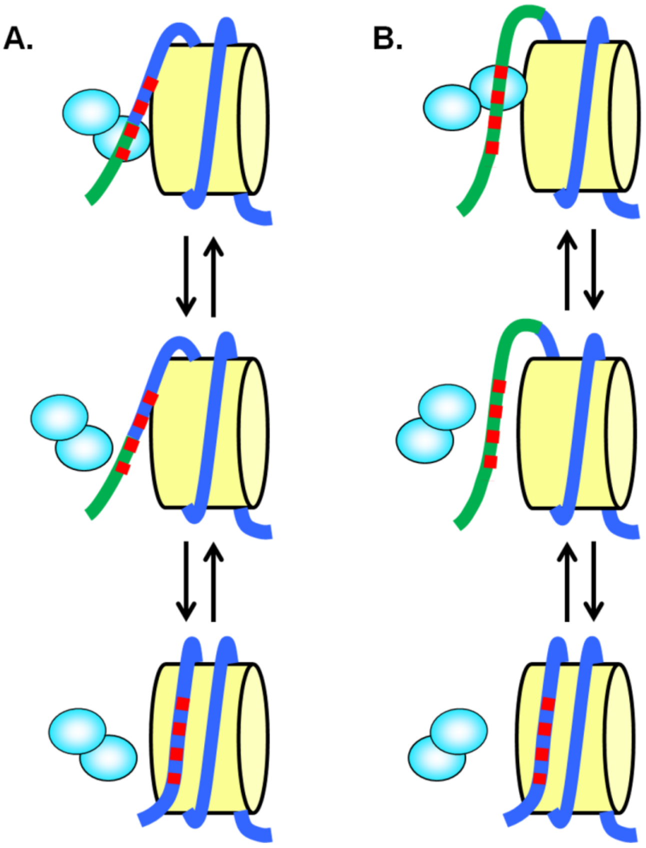

Supplementary Figure 1. Dimeric model of LexA dissociation from a nucleosome when half of the binding site near the nucleosome dyad (A) and half of the binding site far from the nucleosome dyad (B) is mutated. Once the only binding site of LexA dissociates, the nucleosome will be wrapped and only 3 states are included.

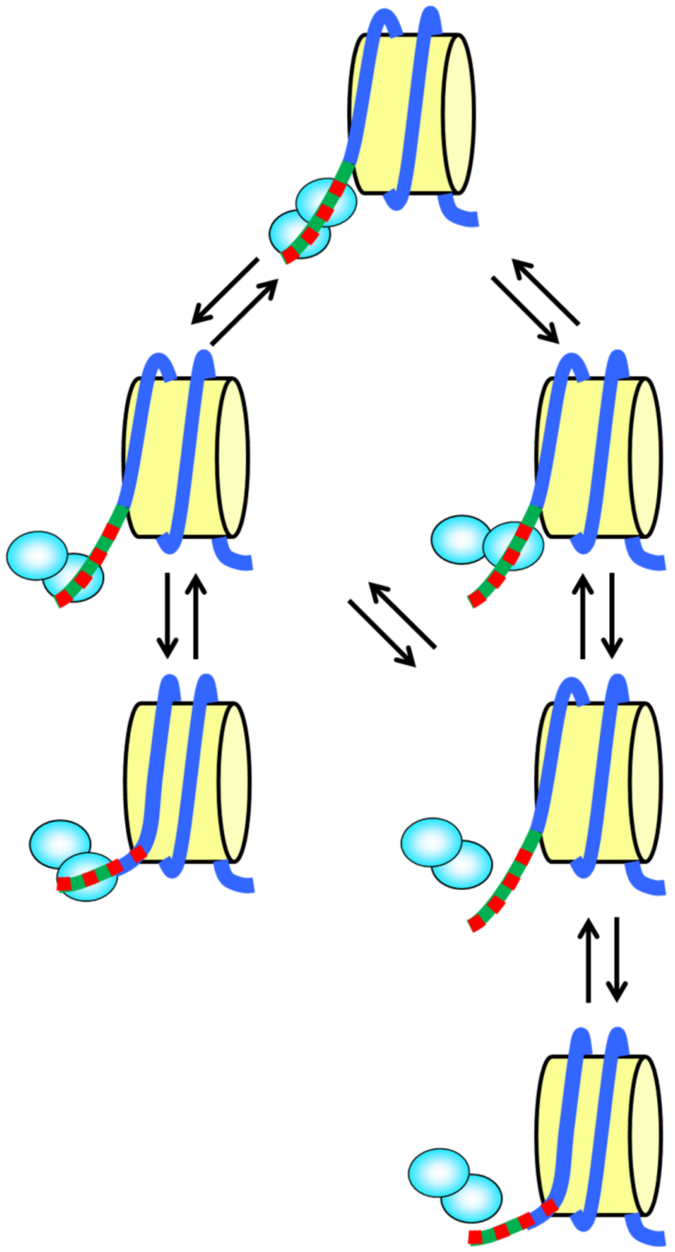

Supplementary Figure 2. Dimeric model of LexA dissociation from a nucleosome when half of the binding site is located outside of the nucleosome positioning sequence. When the LexA binding site is located from positions -10 to +10 of the positioning sequence, the nucleosome cannot unwrap or rewrap in the outside region of the half binding site (-10 to -1). The resulting six state dimeric binding model is indicated.

References

1. Forties, R.A., North, J.A., Javaid, S., Tabbaa, O.P., Fishel, R., Poirier, M.G. and Bundschuh, R. (2011) A quantitative model of nucleosome dynamics. *Nucleic acids research*, **39**, 8306-8313.
